# Can Environmental Regulation Promote Green Innovation and Productivity? The Moderating Role of Government Interventions in Urban China

**DOI:** 10.3390/ijerph192113974

**Published:** 2022-10-27

**Authors:** Yuanshuo Xu, Jiahe Liang, Zhaoyingzi Dong, Minjun Shi

**Affiliations:** School of Public Affairs, Zhejiang University, Hangzhou 310058, China

**Keywords:** porter hypothesis, green innovation, productivity, environmental regulation, local government interventions, environmental policy mixes

## Abstract

Can environmental regulation promote green innovation and the productivity of cities? The “Compliance Cost” (CC) perspective and the “Porter Hypothesis” (PH) offer contrasting views, whereas the existing empirical results are inconclusive. This paper aims to highlight the roles of multifaceted government interventions, including government-to-firm subsidies, tax levies on firms, and environmental infrastructure provisions, in moderating environmental regulation for realizing PH. Based on the fixed-effects models for Chinese prefecture cities from 2005–2013, we found that environmental regulation positively impacted green innovation but negatively affected productivity. The results of moderating effects suggest that environmental regulation can better promote green innovation if it is compounded with more government-to-firm subsidies, lower firm tax burdens, and increased environmental infrastructure provisions. We further decomposed the impacts of these interventions across seven fields of green innovation and found that subsidy and tax burden relief were especially effective in facilitating more GI in the sector of transportation and alternative energy production. This paper amplifies the theoretical framework of PH by accentuating the analytical lens of multifaceted government interventions but also provides insights into how local governments can effectively design “carrot-and-stick” policies to realize PH at the city level.

## 1. Introduction

Over the past four decades, China has experienced rapid economic growth while posing severe problems of environmental degradation [1]. Recently, high-quality development has been imperative for China’s urbanization [2]. How to promote sustainable development is an important issue that requires urgent attention [3]. Within a variety of pathways, promoting green innovation (GI) (Different from normal innovation, green innovation (GI) refers to the technological innovations in products, services, management and process with less or zero negative effects on the environment, which involves areas of energy-saving, pollution-prevention, recycling, and environmental management) is envisaged as one pivotal strategy to drive urban economic growth in a green and innovative way [4]. There is a strong consensus that cities need to have well-designed environmental regulations and multifaceted government interventions to stimulate more efforts and investments in GI [5,6]. Moreover, scholars suggest that arranging appropriate policy mixes is more effective in stimulating GI than adopting a single policy tool [7]. However, few scholarly works have differentiated innovation in GI and non-GI and assessed the compound effects of multi-dimensional policy mixes on GI, especially at the city level. This results in a lack of an integrated roadmap on how local governments can effectively design and combine policies to facilitate GI. This article attempts to fill this gap in the research.

Environmental regulation (ER) (Environmental regulation (ER) refers to the rule and administrative action enforced by the government to control pollution and supervise industrial production to protect the environment) is regarded as a fundamental policy factor to inflect GI [8]. Local governments often employ ER to constrain pollution from firms [9] and, meanwhile, stimulate long-term innovation and productivity growth [10]. Yet, the substantial effects of ER policies are undetermined—two opposite theses of the “Compliance Cost” and “Porter Hypothesis” (PH) have been developed [11]. The cost-driven perspective argues that ER will increase the ‘compliance cost’ from pollution controls and the burden on firms, thereby crowding out investments in innovation and undermining productivity. In contrast, PH suggests that well-designed ER can motivate the innovation of enterprises (weak version) [12] and industrial upgrading for higher productivity (strong version) [13]. The regulated enterprises are expected to increase their inputs in innovation aimed at improving production efficiency to offset “compliance costs” and maximize long-term benefits. Nevertheless, whether PH can be realized is controversial, and the existing empirical results are inconclusive [14]. Furthermore, most studies have not differentiated overall innovation based on the technical fields [15,16]. Therefore, our study differentiates broad innovation into sorts of GI and non-GI. The GI has been further decomposed into seven different fields, which include transportation, waste management, energy conservation, alternative energy production, administration, regulatory or design, agriculture or forestry and nuclear power. We construct panel models for Chinese prefecture cities to investigate the relationships between ER and different fields of GI and productivity to contribute new empirical insights to the ongoing debate.

Recent studies have confirmed that ER has compounded with different local contextual factors of socio-economic features, urban policies and institutional settings to influence green innovation [17]. Among these compound factors, local government interventions are assumed to have the potential to alleviate the negative impacts of ER [18] and yield innovation-induced effects on GI [19]. In practice, local governments often employ a mix of policies consisting of ER and other interventions, e.g., subsidies, tax burden relief and investment in urban environmental infrastructure [20,21,22]. The aim is to create an environment of both regulatory restriction and economic incentives to stimulate the green transformation of firms [19] and ultimately drive GI and productivity growth of cities. Nevertheless, studies are scant to systematically explore the roles of multifaceted government interventions and how they moderate the effects of ER on GI [18]. Our study fills this gap by examining whether and which types of local government interventions are able to moderate ER to help realize PH. The relationships between ER, multifaceted government interventions, and GI or productivity, are the core focus of this paper.

In China, local governments act as “place-based leadership” in pursuit of urban transition for sustainability [23]. As such, urban China provides an excellent ground to testify to the role of local government interventions in actualizing PH. Since the early 2000s, Chinese local governments have imposed considerable production constraints on firms via ER. According to Liu et al. [24], 8210 environmental laws and regulations have been issued since 1970, and nearly 40% of them were published after 2007. Moreover, the “carrot-and-stick” approaches of policy mixes have been widely adopted by local governments to facilitate GI and industrial upgrading. For instance, 10% of the investment in energy-saving and water-saving equipment has been credited to the deduction of business income tax since 2008 [25]. Since 2011, every ton of saved coal has been given a lump-sum subsidy of RMB 240–300 to energy-saving firms [26]. In addition, investing in urban environmental infrastructure has become another important strategy because high-quality public facilities enhance the pollution treatment capacities of cities (e.g., sewage disposal and solid waste treatment) to relieve the compliance burden of firms [20].

Therefore, these three facets of local interventions are examined in this study regarding how they moderate the association between ER and GI. We have answered the following research questions: Can environmental regulation promote green innovation and productivity? Which type of local government intervention is able to positively moderate ER’s effects for the realization of PH? Are the effects of ER, multifaceted government interventions, and their mixes contingent upon the fields of GI to be heterogeneous? This paper contributed with comprehensive fixed-effects models of Chinese prefecture cities from 2005 to 2013 and aimed to investigate the effects of ER, multifaceted local government interventions and their interaction terms on GI and productivity. We decompose the heterogeneous impacts of policy mixes on different fields of GI, which refine our investigations. More importantly, we highlight the moderating roles of multifaceted government interventions of subsidies, firm taxes and environmental infrastructure provisions to amplify the analytical lens of the PH framework and enrich the policy toolbox by incentivizing GI to drive sustainable urban development.

This paper proceeds as follows. The next section reviews the theoretical and empirical literature on PH, focusing on the relationships between ER and GI. Then, it reviews the studies of government interventions and discusses the potential mechanisms of multifaceted governmental regulations in moderating ER. Section three introduces our key variables of interest, their descriptive statistics and model specifications. Section four analyzes the model results with emphasis on the direct effect of ER and the moderating effect of government interventions. These effects are further examined by decomposing GI into seven different fields. The robustness checks are also provided. Finally, the last section discusses the main findings of this study and highlights our contributions and policy implications.

## 2. Literature Review

Previous studies have widely investigated the direct effect of ER on innovation and productivity, while recent scholarly interests have shifted to GI [27]. Local government interventions are regarded as a crucial factor that interplays with ER and affects the realization of PH, but studies that systematically examine the moderating role of multifaceted government interventions are scant [18]. Analyzing the heterogeneous impacts of ER and its compound effects with various government interventions on different technical fields of GI is novel and suggestive [15].

### 2.1. The Porter Hypothesis: The Developmental Impacts of Environmental Regulation

PH argues that a well-designed ER can promote innovation (weak version) and productivity (strong version) [11]. The rationales are threefold. First, the appropriate ER would prompt firms to reduce pollution-intensive production and motivate them to offset the ‘compliance cost’ via technological innovation and industrial upgrading [28]. Second, the well-crafted ER can correct the negative externality of environmental pollution by internalizing the pollution costs of enterprises [5]. Third, ER is also expected to drive pollution-intensive industries to shut down or relocate, encouraging the formation of environmentally-friendly enterprises [29].

Empirics on whether ER can realize strong and weak versions of PH are inconclusive. Scholars have found negative [12,18,29], positive [30,31,32] and non-significant effects [15,33] of ER on productivity and innovation. Few studies have testified on both versions of PH simultaneously [13], while the effect of ER on innovation and productivity might be different. Moreover, research without differentiating the types of innovations might be misleading because not all of the innovations are environmentally friendly and can be induced by ER [14,34]. Nevertheless, due to the data limits, earlier studies rarely categorized innovation when they examined ER’s effects [20]. Until recently, studies have shifted to the relationship between ER and GI, but empirical results are still mixed [22]. Aside from the positive effects [9,35], studies have also revealed the negative [36,37] and non-significant impacts [38] of ER on GI. For instance, Zhang et al. [36] have found that the regulation of carbon emission crowds out GI from enterprises in China, and this inhibitory effect is stronger in the eastern regions and places with low-emission intensity. By contrast, using a dataset of Chinese industrial sectors from 2003 to 2010, Hu et al. [35] have shown that ER can promote GI in manufacturing sectors with higher foreign investment. Moreover, the differential effects of ER have been attributed to the types of ER [39]. For example, according to Feng and Chen [39], the administrative ER has a positive effect, while market-based ER is likely to play a negative role on green industrial development. The ER approach that relies on public participation has a non-significant effect [39]. Using the survey data from 298 high-end manufacturing firms in China, Zhang et al. [28] have confirmed that both the command-and-control and the market-based types of ER can increase firms’ intentions to pursue GI, whereas their impacts are heterogeneous across end-of-pipe innovation, cleaner process innovation and green product innovation.

Additionally, most of these works are conducted at the industrial [35], provincial [19,22] or firm level for listed companies [30], while the city-level studies are limited [27]. Additionally, they tend to rely on subjective data (e.g., survey data [28]) and indirect measures (e.g., energy consumption [19] and R&D expenditures [39]) to quantify innovations, which are hard to capture the actual innovation performance of enterprises. Furthermore, few works have systematically considered the effects of policy mixes, especially for the potential moderating effects on ER [21]. In addition, different sectors may have distinctive determinants and driving mechanisms on GI [15], whereas very few studies have decomposed GI based on technical fields and analyzed the possible heterogeneous impacts of ER. For instance, based on the panel data of 33 countries over the 1990–2015 period, scholars have found that stringent ER tended to induce more GI for marine, geothermal and hydropower but exerted an insignificant effect on GI of solar and wind energies [40]. The ER in the UK is able to stimulate effective end-of-pipeline technologies but has no effect on cleaner production technologies [15]. In China, ER has a positive effect on cleaner processes and green product innovation, but this effect is not significant on end-of-pipe innovation [28]. Therefore, decomposing GI by sectors is important in refining the investigation of PH and the innovation-induced effects of ER [41,42].

### 2.2. Government Intervention: The Moderation Factor of the Porter Hypothesis

Local governments are responsible for environmental protection and urban development [18]. Their multifaceted policy interventions interconnect with ER and co-shape the choices of firms among compliance, innovation and industrial upgrading [18]. In China, local governments are enthusiastic about implementing the “carrot-and-stick” approaches of multi-pronged policy mixes [43]. The aim is to compensate the regulated firms and support their pursuits on GI and industrial upgrading. These policy tools often include financial subsidies [22,40], tax burden reliefs [21] and provisions of environmental infrastructure [19,44]. Two possible driving mechanisms are discussed here.

First, the knowledge and environmental spillovers of GI are believed to yield insufficient market incentives for firms to pursue GI [14,15]. On the one hand, firms have concerns about the free-rider problem due to the “positive externality of knowledge and environmental benefits.” This undermines their efforts in GI [12]. On the other hand, investing in GI and industrial upgrading is costly and risky because the returns are often uncertain [44]. By providing a variety of subsidies and tax refunds, local governments can internalize the double externalities and lower the risk of firms, which facilitates the realization of the Porter effects [18].

Second, the high capacity of urban environmental infrastructures (UEI) can share the cost of firms’ pollutant treatment (e.g., solid waste, industrial smoke and sewage) and relieve their stresses under ER [45,46]. Urban environmental infrastructure is mainly funded by local government for ecological enhancement and pollution abatement, including sewage treatment facilities, waste treatment facilities, industrial waste disposal systems, energy infrastructure and pollution control systems [45]. The substitutive effect of UEI, with respect to pollution treatment, allows firms to save financial resources for GI and productivity growth [18]. Moreover, GI and UEI can be mutually reinforced because some green innovations are specifically designed to improve UEI [47]. Moreover, cities that embrace the high capacity of UEI may lower their standards of ER since the abatements of pollutants can be achieved through UEI.

Empirical studies also indicate inconsistent results regarding whether multifaceted government interventions can moderate ER for the realization of PH. For example, using the panel data of 30 Chinese provinces from 2009 to 2015, Guo et al. [19] have found that ER inhibits GI, but local government’s investments in R&D activities can positively moderate the association between ER and GI. In a study of 11 provinces along the Yangtze River Economic Belt in China, both the government R&D subsidies and ER increase the GI efficiency of the manufacturing industry; however, the moderating effects of R&D subsidies are not significant [48]. For the strong version of PH, its realization can be moderated by the R&D investment [49], while Song et al. [21] suggest that tax incentives can help realize the weak version of PH. Zhou et al. [18] have pointed out that the interaction term of ER and subsidies negatively impacts the productivity of firms. By contrast, Wang et al. [22] have found that the subsidies from the provincial government in China positively moderate the GI of firms, but the marginal effect decreases when subsidies are above a certain level. Additionally, Costantini et al. [7] suggest that a more balanced use of demand-pull and technology-push instruments yields significant innovation-induced effects.

### 2.3. Research Trends and Innovation of This Study

Previous studies on PH have focused on the direct effect of ER on overall innovation and productivity, while recent investigations have shifted their focus to GI and highlighted the importance of taking into account various local government interventions together with ER. The co-examination for the moderating roles of multifaceted government interventions in both versions of PH is limited, and the heterogenous impacts of ER and government interventions on different sorts of GI have been largely overlooked. In this paper, we systematically analyze the roles of “carrot-and-stick” approaches comprising ERs, subsidies, tax reliefs and the provisions of urban environmental infrastructure at the city level. We also decompose GI into seven sorts and delve into the heterogenous effects. This paper seeks to amplify the PH framework and enrich the local government’s policy toolbox for sustainable urban development.

## 3. Materials and Methods

### 3.1. Data Sources and Variables Selection

#### 3.1.1. Data Sources

Our data included 270 cities at the prefecture level and above in China (We excluded 16 prefecture cities in our sample since there is no available data). Prior studies suggest that a city would be a pivotal unit of analysis because it is the most relevant contributor to promoting economic growth and addressing pollution abatement [27,50]. Chinese local governments also play significant roles in interventions at the city scale. A city is also a unit with stable boundaries that enable studies over time.

Four different sources of data from 2005 to 2013 were collected and aggregated at the prefecture city level to construct model variables (The sample in 2009 was excluded due to data availability. To ensure data integrity, we conducted an analysis using data from 2005 to 2008 and 2010 to 2013). First, we collected patent data to measure GI from the IncoPat global patent database, which covers enormous amounts of detailed patent information. Second, we retrieved the pollutant removal data from the China City Statistical Yearbook to quantify the stringency of ER. We also collected the socio-economic data for calculating the total factor productivity and measuring all of our control variables based on the China City Statistical Yearbook. Third, to measure the multifaceted government interventions, we relied on the Annual Survey of Industrial Firms (ASIF) database from the China National Bureau of Statistics. This database covers enterprises with annual sales above RMB 5 million, accounting for 95% of the production output in China. It provides firm-level information, including the geo-location of firms. Based on the sequential identification method, the total output values, the received subsidies and tax burdens of firms were obtained for the construction of two variables: government subsidy and tax burdens. Last, using the wastewater treatment data from the China Construction Statistical Yearbook, we measured the capacity of urban environmental infrastructure as the proxy of the government’s capacity for pollution treatment services. 

#### 3.1.2. Dependent Variables

- Green Innovations (GI)

Scholars have identified patent data as a reliable indicator to reflect innovation performance [51], which has been adopted in recent studies to measure GI [27,31]. Compared with other potential candidates of indicators (e.g., R&D expenditures, number of researchers), patent data is a more direct measure of innovation [52]. In this study, we differentiated green and non-green patents based on the four-digit code of International Patent Classification (IPC) from the World Intellectual Property Organization (WIPO). According to the “IPC Green Inventory”, as listed by the “United Nations Framework Convention on Climate Change (UNFCC)”, we further decomposed general green patents into seven sub-categories, embodying fields of transportation, waste management, energy conservation, alternative energy production, administrative, regulatory or design, agriculture or forestry and nuclear power.

- Productivity (TFP)

We employed the Stochastic Frontier Analysis (SFA) method [53] to calculate total factor productivity (TFP) for each city. The SFA method divides actual output into three parts: production function, random factors and technical inefficiency. The SFA considers the effect of random factors on actual output so it can get a more stable estimation than other prevailing methods, such as the Data Envelope Analysis (DEA), which completely ascribes technical efficiency as the difference between actual output and frontier output. Other studies on the examination of PH have used this method to measure productivity [54]. Specifically, the inputs are measured by the amount of labor population and fixed assets, and the real GDP is used to represent the output. Regarding the value of VIF, we selected the Cobb-Douglas production as the Stochastic Frontier function to estimate parameters. The formulas are as follows:

(1) $$lnY_{it}=\beta_{0}+\beta_{1}ln\left(L_{it}\right)+\beta_{2}ln\left(K_{it}\right)+\left(v_{it}-u_{it}\right)$$

where *i* denotes the city and *t* denotes the year. *Y* refers to real GDP. $L_{it}$ is the amount of labor population. $K_{it}$ is a fixed asset. $v_{it}$ is the random error term. $u_{it}$ is the technical inefficiency term. The value of technical efficiency is calculated as the ratio of actual output expectations to frontier output expectations, namely:

(2) $$TE_{it}=\frac{E\left[f\left(X_{it},\;t\right)exp\left(v_{it}-u_{it}\right)\right]}{E[f\left(X_{it},\;t\right)exp\left(v_{it}\right)|u_{it}=0]}=exp\left(-u_{it}\right)$$

$TE_{it}$ reflects the status of technical efficiency of the city *i* in year *t*. When $u_{it}$ = 0,$\;TE_{it}=1$, which represents technical efficiency; if $u_{it}>0$, $TE_{it}<1$, which denotes technical inefficiency.

#### 3.1.3. Independent Variables

- Environmental Regulations Stringency (ER)

Following the previous literature [27,55], we utilized the entropy method to construct a comprehensive index to measure the full picture of environmental regulation stringency in cities. This approach avoids bias in the single indicator method or subjectivity in the scoring criteria method [56]. We rely on five treatment indicators to capture the pollution controls, including the comprehensive utilization rate of industrial solid waste, the removal rate of SO2, the removal rate of industrial smoke (powder) dust, the harmless treatment rate of domestic garbage and the treatment rate of city sewage. The value of ER ranges from 0 to 1, and a higher value implies stricter regulation. The calculation process of the entropy weight of ER is as follows:

1. Standardize each indicator:

(3) $$X_{ijt}^{*}=\frac{X_{ijt}-min\left(X_{jt}\right)}{max\left(X_{jt}\right)-min\left(X_{jt}\right)}$$

where $X_{ijt}$ is the indicators *j* of the city *i* at year *t*, and $max\left(X_{jt}\right)-min\left(X_{jt}\right)$ are the largest and smallest values of indicator *j* at year *t*.

2. Utilize the entropy method to determine the weight of each indicator:

(4) $$e_{jt}=-\frac{1}{log\left(m\right)}\Sigma_{i=1}^{m}y_{ijt}log(y_{ijt})$$

where $y_{ijt}=\frac{X_{ijt}^{*}+1}{\Sigma_{i=1}^{m}\left(X_{ijt}^{*}+1\right)}$ is the contribution of each standardized indicator at time *t*.

3. Calculate the divergence coefficient:

(5) $$d_{jt}=1-e_{jt}$$

4. The weight of indicator *j* at time *t*:

(6) $$W_{jt}=\frac{d_{jt}}{\Sigma_{j=1}^{n}d_{jt}}$$

5. Calculate the urban environmental regulation stringency index, ER:

(7) $$ER_{it}=\Sigma_{j=1}^{n}W_{j}X_{ijt}^{*}$$

- Multifaceted Government Interventions

Our core interest is to examine the moderating roles of multifaceted government interventions. We choose three variables to represent the local interventions: (1) government subsidy (SUB), (2) tax burdens (TAX), and (3) the capacity of urban environmental infrastructure (UEI). Government subsidy is the most common and direct support offered by the local government, including a variety of fund schemes (e.g., innovation and technology fund and green transformation fund) and financial supports (e.g., energy-saving and renewable energy subsidies) [18]. Local governments can also adjust the firm’s tax burdens by designing tax rates, tax preference policies and refund systems. By June 2019, Chinese cities have introduced 89 tax preferences [21]. The provision of UEI also matters. Previous studies pointed out that the establishment of UEI (such as centralized wastewater treatment plans and solvent recovery systems) can significantly lower per-unit treatment costs of firms’ pollutants [46] and exert a positive moderating effect for realizing PH [18].

1. Government subsidy (SUB)

The major financial instrument of local government is the government-to-firm subsidy. Following the measurement by Zhou et al. [18], the ratio of the government subsidies a firm has received to its total output value was used to measure the financial support of local government. The higher ratio indicates more financial support from governments.

2. Tax burdens (TAX)

According to China’s major categories of local business taxes, three major tax levies of business taxes, value-added taxes, and enterprise income taxes were aggregated to capture firms’ tax burdens. The firm’s total output was further normalized by the tax burdens.

3. Urban environmental infrastructure (UEI) capacity

In a recent examination of PH, Zhou et al. [18] constructed a dummy variable of industrial zone establishment for measuring the local government’s effort in providing environmental infrastructure. However, this measurement is industrial-zone based and may not be able to reveal the different levels of UEI capacity across cities. Therefore, we captured the UEI capacity by adding the total quantity of treated wastewater and the quantity of recycled and reused wastewater (10,000 m^3^) and divided it by the quantity of discharged wastewater (10,000 m^3^). The higher value of UEI represents a higher capacity of urban environmental infrastructure.

- Control variables

Following the existing empirics, we controlled for foreign direct investment per capita (FDIPC) [27], population density (POP_DEN) [57], the number of industrial enterprises above the designated size (FIRM) [58], and the proportion of employees in the secondary industry (EMPLOY) [59]. Foreign direct investment may bring advanced technology to drive more GI and higher productivity to cities. The population size and the number of industrial enterprises are expected to be positive predictors. An industrial structure may also lead to different responses of firms to ER.

Table 1 offers the mean and standard deviation for all variables that are utilized in this paper. The high standard deviation of *GPAT* shows a great discrepancy of green patents across cities. The mean values of seven types of GI reflect the between-heterogeneity; therein, the green patents are largely concentrated in the technical field of alternative energy production (*ALTER*), waste management (*WASTE*) and energy conservation (*ENERGY*). The mean value of *TFP* was 1.39. The average *ER* was 0.65. Concerning government interventions, the average ratio of government subsidies to the aggregated total output value of firms was 0.27%. The average tax burden of firms to the aggregated total output value of firms was 6.20%. The mean value of *UEI* showed that the urban environmental infrastructure addressed 74.57% of pollutants on average.

All data is reported in original form. *GPAT*, *SUB*, *TAX* and *UEI* have transformed to natural logarithm form when used in the regression models; *GPAT* = overall green patents. *GPAT* is decomposed into the fields of transportation (*TRANSPORT*), waste management (*WASTE*), energy conservation (*ENERGY*), alternative energy production (*ALTER*), administrative, regulatory or design (*DESIGN*), agriculture or forestry (*AGRI*) and nuclear power generation (*NUCLEAR*); *FDIPC* = Direct foreign investment (RMD 1000 per 10,000 people); *POP_DEN* = Total population (thousand) per km^2^; *FIRM* = Number (thousand) of industrial enterprise above designated size; ASIF = China’s Annual Survey of Industrial Firms.

### 3.2. Model Specification

The panel regression model was conducted to examine the impacts of ER on the types of GI and productivity. The Hausman test based on the Wald criterion showed the appropriateness of the fixed-effects models in the present work. We introduced three interaction terms between *ER* stringency and other government intervention variables to test the moderating effects of these interventions on the association between *ER* and GI or productivity.

(8) $$Y_{i,\;t}=\alpha_{0}+\beta_{1}ER_{i,t}+\beta_{2}\left(ER_{i,t}*SUB_{i,t}\right)+\beta_{3}\left(ER_{i,t}*TAX_{i,t}\right)+\beta_{4}\left(ER_{i,t}*UEI_{i,t}\right)+\beta_{5}SUB_{i,t}+\beta_{6}TAX_{i,t}+\;\beta_{7}UEI_{i,t}+\sum_{j=1}^{k}\beta_{j}X_{i,t}+\eta_{i}+u_{t}+\varepsilon_{i,t}\;$$

where *i* denotes the city and *t* denotes the year. $Y_{i,\;t}$ refers to dependent variables: green innovation (log-form), green innovation by fields and productivity in the city *i* and year *t*. $ER_{i,t}$ is the stringency measure of environmental regulation in the city *i*. $SUB_{i,t}$ and $TAX_{i,t}$ refers to the total government subsidies (natural logarithm-form) and tax burdens (natural logarithm-form) of firms in the city *i* and year *t*, respectively. $UEI_{i,t}$ represents the capacity of urban environmental infrastructure. $X_{i,t}$ are control variables, including foreign direct investment per capita (*FDIPC*), population density (*POP_DEN*), the number of industrial enterprises above designated size (*FIRM*) and the proportion of employees in the secondary industry (*EMPLOY*). $\alpha_{0}$ is the constant term. We included the fixed effects of time $\eta_{i}$ for the control of possible external shocks over time and the fixed effects of city $u_{t}$ for the control of unobserved city-specific factors. ε is the error term.

We expect GI and productivity to be influenced by ER, government interventions and their compounded effects. We hypothesize that stricter ER inhibits GI and productivity due to the rising compliance costs of pollution control. Government subsidy and tax burden relief can financially alleviate the negative impact on GI due to compliance costs. The well-developed UEI is assumed to help firms reduce compliance costs by substituting the firms’ efforts on pollution abatement with the service provisions of public infrastructure. Therefore, we expect that “carrot-and-stick” policy mixes are able to enhance GI and productivity. The heterogeneous impacts of ER and other government interventions on GI have been examined across different fields.

## 4. Results

Table 2 shows the panel regression results. Models (1) and (2) are the estimations of the direct effects of ER and local government interventions on GI and productivity. Models (3) and (4) testify to the compound effects of policy mixes. We ran the models using robust standard errors. Our models give a comprehensive picture of how the multifaceted local government interventions interact with ER to co-shape GI and productivity in cities. In our models (1) and (2), ER had a positive and significant correlation with GI and a negative impact on productivity. This supports the weak version of PH that the ER stimulates GI, but the implementation of ER may undermine productivity. There may exist a gap in transferring the induced innovations into “innovation compensation”. Additionally, the comparison of adjusted R^2^ indicates that we have better model performance on GI than productivity, thus solidifying our proof of the weak PH.

### 4.1. The Direct Effects of Multifaceted Government Intervention

Regarding the direct effects of government interventions, both coefficients of SUB were negative. The firms that rely on government subsidies were less efficient in producing GI and promoting industrial upgrading. TAX had no direct impact on either GI or productivity. A previous study suggests that tax burden relief might only spur product sales but fails to correct the externality problem in innovation-driven industrial upgrading [60]. The improvement of UEI enhanced GI but not for productivity. The well-developed UEI could free up firms’ resources by offering cost-effective public services for pollution treatment. Local governments would also procure firms’ GI in upgrading UEI and, therefore, incentivize firms to pursue GI.

### 4.2. The Moderating Effects of Multifaceted Government Intervention

The coefficients of ER in models (3) and (4) show that the innovation-induced effect of ER has been strengthened, and the negative impact of ER on productivity has been offset after including three variables of interventions. Compared with the sole implementation of ER, the adoptions of multiple interventions comprising “carrot-and-stick” approaches were more effective in relieving the stress from ER and triggering the Porter effect.

Table 2 shows that the interaction term of subsidy with ER is critical in driving GI, but ER × SUB is not associated with productivity. Cities with stringent ER can stimulate more GI when more government subsidies are given to firms. Subsidies provide financial incentives and enhance capacities for regulated firms to pursue GI as the response to ER. Another interaction term, ER × TAX, negatively affected GI. Therefore, lowering the tax burden of firms is another option for local governments to moderate the association between ER and GI for the realization of the Porter effect. According to the coefficient of TAX in Model (3), the local government should not solely rely on the reduction of the firm’s tax burdens while ER is in place. Rather, it should design an appropriate tax policy that acts in concert with ER to ensure the “carrot-and-stick” approach is effective.

Alternatively, to motivate firms in GI, cities could also choose to enhance the capacity of environmental infrastructures to facilitate the innovation-induced effects of ER. According to Table 2, ER × UEI had a positive effect on GI but negatively correlated with productivity. Under this circumstance, firms are more incentivized to respond with GI for long-term benefits rather than invest in expensive environmental equipment for higher productivity to cope with ER. Notably, the coefficient of UEI in Model (3) is negative, which shows that the provision of UEI needs to compound with ER. This suggests that the impact of the “carrot-and-stick” approach is larger than a single-policy instrument.

In terms of control variables, green innovation is higher in cities with more direct foreign investment. This is consistent with the theory of the “pollution halo” [61], even though foreign direct investment may not directly benefit productivity. The cities that had higher population densities, more industrial firms and a higher percentage of secondary industry tended to have more GI.

### 4.3. The Heterogenous Effects of Multifaceted Government Intervention

We further decomposed GPAT into seven fields and ran the regression models to examine the heterogenous effects of ER. The sub-model results are presented in Table 3 and Table 4. The first set of models (Models (5) to (11)) shows the direct effects, and the second set of models (Models (12) to (18)) captures the compound effects of ER and local government interventions. The sub-model estimations control for the same socio-economic variables, ensuring that the results are comparable to the overall models. The coefficients reflect the heterogeneous impacts of multifaceted government interventions on GI regarding different technical fields.

The ER of cities positively correlated with GI in transportation, waste management, energy conservation, alternative energy production, administration, regulatory or design and agriculture or forestry, but it had no significant effect on GI in the field of nuclear power. Similar to the overall model, the subsidy itself had negative effects on all sub-fields of GI. Higher tax burdens were positively associated with GI in the fields of administration, regulatory or design and nuclear power. Cities with higher capacity of UEI were featured with more GI in waste management and energy conservation.

The moderating effects of the interaction terms between ER and multifaceted government interventions are evident in the sub-models. For example, ER × SUB motivates the GI on alternative energy production, whereas it does not affect other fields of GI. Reducing the tax burden could positively moderate ER’s impacts on GI in transportation and alternative energy production. Furthermore, the moderating effects of UEI were found in most fields of GI except for nuclear power, suggesting that increasing environmental infrastructure provisions can be an effective policy tool to compound ER for the realization of the Porter effect.

### 4.4. Robustness Check

As for the robustness check, this paper re-estimated models (1) and (3) by replacing our main dependent variable. We replaced GI (measured by authorized green patent numbers) with non-green innovation (NGI) (measured by non-green patent numbers) and overall innovation (OI) (measured by all authorized patent numbers). We ran the fixed-effects models using robust standard errors. The results are shown in Table 5. Compared with the results presented above, these model results only produced minor changes. Therefore, our previous conclusion regarding the positive moderating roles of multifaceted government intervention is robust.

## 5. Discussion, Conclusions and Limitations

### 5.1. Discussion

Our analysis of moderating effects provides new insights for PH. Local governments should employ “carrot-and-stick” approaches of policy mixes in stimulating GI and promoting industrial upgrading. Specifically, cities should pay explicit attention to the appropriate combinations of environmental regulation (stick), subsidies (carrot), firm burden relief (carrot) and improvement of infrastructures (carrot). Based on the magnitude of coefficients, we discuss their heterogeneous effects on specific sectors of GI, which include transportation (*TRANSPORT*), waste management (*WASTE*), energy conservation (*ENERGY*) and alternative energy production (*ALTER*), as follows.

To motivate GI in transportation, the local government can relieve the tax burden of firms when ER is in place. If there is a 10% reduction with respect to the ratio of firm taxes to the firm’s total output, then the number of transportation patents will increase by 114.1% on average. In China, numerous deductions and exemptions of the value-added tax have been provided to the manufacturer of new-energy vehicles since the 2010s, which manages to encourage firms to pursue innovation [62]. For promoting GI in the field of alternative energy production, ER should be implemented with both firm subsidies and tax relief. If the government subsidy increases by 10% while ER is in place, the number of alternative energy production patents will increase by 45.5% on average. This result resonates with several subsidy strategies of the Chinese government, which primarily aim at supporting the new energy sector, such as the Energy Saving and Emission Reduction Plan (ESERP) in 2006. A 10% tax rate decrease can also motivate innovations in the field of alternative energy production when patents increased by 146.8%. Importantly, these four sorts of GI can be significantly driven by the policy mixes of ER and the provision of UEI with high capacity. Under the same stringency of ER, a 10% increase of the UEI capacity can motivate these fields of GI patents ranging from 30.1% to 56.8% on average.

Our findings have implications for policymakers and urban planners who seek an efficient policy toolbox for sustainable urban development. Regarding the implementation of fiscal and tax policies, we suggest that local government should reduce direct grants to firms but increase subsidies and lower the tax burdens when ER is in the precondition. The “stick” policy of ER pushes the subsidized firms to invest in green innovations. For instance, China’s low-carbon pilot city program has effectively promoted green transformation through the strategic move of setting environmental standards, providing green subsidies and reducing tax burdens for firms [63]. The provision of urban environmental infrastructure is an effective means to moderate the innovation-induced effect positively. For example, the “Sponge City” agenda and the “Zero-Waste City” program in China were initiated to underpin green development for embedded energy-saving and recyclable technology in urban environmental infrastructures [64,65].

### 5.2. Conclusions

In this paper, our analysis validates the co-existence of the weak PH and the “compliance cost” in urban China by conducting panel models at the city level. The “innovation-induced” effect is strengthened, and the negative impact of ER on productivity can be offset if local governments adopt multi-pronged interventions comprising “carrot-and-stick” approaches rather than solely implementing ER. We confirm that GI is higher in cities if ER interconnects with more government-to-firm subsidies, lower tax levies, and higher capacity in urban environmental infrastructure. We further investigate the heterogenous impacts of policy mixes on different fields of GI to polish the framework of PH. Specifically, fiscal policies of subsidy and tax burden relief are effective in facilitating more GI in the sector of transportation and alternative energy production. The provision of UEI is an effective means to compound with ER for triggering the innovation-induced effects of ER.

Our analysis contributes to scholars’ call for the systematical investigation of multifaceted government interventions to enrich the framework of PH [18]. We contribute to the ongoing debates on the impacts of ER by not only testifying on the weak and strong versions of PH but also by showing that, in the state-centric context of development and governance (such as in China), the well-designed local government interventions and their strategic combinations are essential for promoting GI and productivity. Moreover, the effectiveness of ER and the compound effects with other interventions rests with the specific fields of GI. As far as we know, this is the first analysis that makes a particular effort to decompose GI and differentiate the main drivers across various fields of GI. This new nuanced understanding could provide implications for local governments when they struggle with environmental protection and economic development.

### 5.3. Limitations and Prospects

We realize the potential limitations of this study. First, the indicator we chose to measure ER needs to be improved. Although our entropy-based index can capture the complete picture of ER stringency, this measurement does not allow us to decompose the impact of heterogeneous ER on GI and productivity (e.g., command-and-control regulation, market-based regulation and voluntary regulation). Second, our theoretical framework needs to be expanded. This study only covered three facets of government interventions, while other policy tools may also play a moderating role to help realize PH, such as a green credit policy, government green bond and R&D investment. Third, this paper only examined the moderating role of government interventions, while the mechanisms underlying the interaction between ER, government interventions and GI or productivity may be more complex and spatially varied. Therefore, we call for more empirical studies employing qualitative methods or spatial models to enrich the stories of multifaceted government interventions within the framework of PH. A more fine-grained analysis of the role of heterogeneous ER and other facets of government interventions is the direction for future research.

## Figures and Tables

**Table 1:** Descriptive Statistics of Model Variables.

Variables		Data Source	Mean	Std. Dev.
Green innovations	*GPAT*	IncoPat Global Database	128.92	673.37
	*TRANSPORT*		4.86	18.91
	*WASTE*		37.99	180.09
	*ENERGY*		32.74	199.40
	*ALTER*		43.79	246.94
	*DESIGN*		3.60	32.93
	*AGRI*		5.40	21.95
	*NUCLEAR*		0.61	6.54
Productivity	TFP	China City Statistical Yearbook	1.39	0.75
Government intervention	ER	China City Statistical Yearbook	0.65	0.15
	SUB	ASIF	0.27%	0.63%
	TAX	ASIF	6.20%	3.34%
	UEI	China Urban Construction Statistical Yearbook	74.57%	31.84%
Control Variables	FDIPC	China City Statistical Yearbook	0.98	1.75
	POP_DEN		0.44	0.33
	FIRM		1.17	1.60
	EMPLOY		44.98%	13.66%

**Table 2:** Panel regression results for green innovation and productivity.

	(1)	(2)	(3)	(4)
Variables	*GPAT*	*TFP*	*GPAT*	*TFP*
*ER*	3.590 ***	−1.603 ***	3.952 *	−1.669
	(0.325)	(0.190)	(2.254)	(1.213)
*ER* × *SUB*			0.453 **	0.0936
			(0.224)	(0.145)
*ER* × *TAX*			−1.057 **	−0.181
			(0.524)	(0.331)
*ER* × *UEI*			0.616 ***	−0.251 **
			(0.151)	(0.104)
*SUB*	−0.319 ***	−0.257 ***	−0.589 ***	−0.319 ***
	(0.0374)	(0.0262)	(0.141)	(0.0945)
*TAX*	−0.120	−0.0817	0.563 *	0.0315
	(0.111)	(0.0754)	(0.327)	(0.223)
*UEI*	0.108 ***	0.00841	−0.192 **	0.129 **
	(0.0196)	(0.0151)	(0.0780)	(0.0548)
*FDIPC*	0.301 ***	−0.0501 **	0.281 ***	−0.0373
	(0.0872)	(0.0252)	(0.0847)	(0.0261)
*POP_DEN*	1.888 **	0.688 ***	1.828 **	0.758 ***
	(0.890)	(0.199)	(0.858)	(0.204)
*FIRM*	0.286 *	−0.417 ***	0.277 *	−0.412 ***
	(0.155)	(0.0794)	(0.152)	(0.0781)
*EMPLOY*	0.0362 ***	−0.0253 ***	0.0353 ***	−0.0245 ***
	(0.00727)	(0.00435)	(0.00711)	(0.00443)
*Constant*	−4.877 ***	1.903 ***	−4.849 ***	1.832 **
	(0.513)	(0.271)	(1.425)	(0.822)
City Fixed Effects	YES	YES	YES	YES
Time Fixed Effects	YES	YES	YES	YES
Observations	1429	1429	1429	1429
R^2^	0.507	0.147	0.519	0.151
Adjusted R^2^	0.504	0.142	0.515	0.144

Robust standard error in parentheses; *** *p* < 0.01, ** *p* < 0.05, * *p* < 0.1.

**Table 3:** Direct effects of government interventions across fields of GI.

	(5)	(6)	(7)	(8)	(9)	(10)	(11)
Variables	*TRANSPORT*	*WASTE*	*ENERGY*	*ALTER*	*DESIGN*	*AGRI*	*NUCLEAR*
*ER*	1.008 ***	3.156 ***	2.315 ***	2.787 ***	0.911 ***	1.844 ***	0.0633
	(0.226)	(0.318)	(0.280)	(0.293)	(0.200)	(0.287)	(0.127)
*ER* × *SUB*							
							
*ER* × *TAX*							
							
*ER* × *UEI*							
							
*SUB*	−0.0924 ***	−0.231 ***	−0.268 ***	−0.262 ***	−0.0422 **	−0.128 ***	−0.0246 **
	(0.0272)	(0.0365)	(0.0349)	(0.0335)	(0.0186)	(0.0260)	(0.0110)
*TAX*	−0.0289	−0.0533	0.0240	−0.134	0.0972 *	0.0444	0.0849 **
	(0.0688)	(0.101)	(0.102)	(0.102)	(0.0547)	(0.0945)	(0.0363)
*UEI*	0.000244	0.0733 ***	0.0103	0.0473 ***	−0.00834	0.0180	−0.00452
	(0.0128)	(0.0187)	(0.0147)	(0.0169)	(0.00855)	(0.0129)	(0.00296)
*FDIPC*	0.296 ***	0.309 ***	0.322 ***	0.291 ***	0.267 ***	0.235 ***	0.122 ***
	(0.0660)	(0.0854)	(0.0939)	(0.0777)	(0.0579)	(0.0702)	(0.0312)
*POP_DEN*	1.662 ***	1.791 **	2.196 **	1.534 *	1.746 ***	2.064 **	1.273 *
	(0.636)	(0.837)	(0.892)	(0.804)	(0.672)	(1.019)	(0.754)
*FIRM*	−0.0385	0.274 *	0.141	0.222	−0.0839	0.0478	−0.0370
	(0.148)	(0.154)	(0.141)	(0.143)	(0.133)	(0.132)	(0.0855)
*EMPLOY*	0.0141 **	0.0325 ***	0.0236 ***	0.0292 ***	0.00880 **	0.0106 *	−0.000335
	(0.00642)	(0.00738)	(0.00735)	(0.00691)	(0.00414)	(0.00619)	(0.00244)
*Constant*	−2.290 ***	−4.399 ***	−4.357 ***	−4.486 ***	−1.515 ***	−2.772 ***	−0.455 *
	(0.408)	(0.495)	(0.501)	(0.474)	(0.344)	(0.517)	(0.276)
City Fixed Effects	YES	YES	YES	YES	YES	YES	YES
Time Fixed Effects	YES	YES	YES	YES	YES	YES	YES
Observations	1429	1429	1429	1429	1429	1429	1429
R^2^	0.228	0.444	0.375	0.439	0.233	0.265	0.122
Adjusted R^2^	0.224	0.441	0.372	0.436	0.229	0.261	0.118

Robust standard error in parentheses; *** *p* < 0.01, ** *p* < 0.05, * *p* < 0.1.

**Table 4:** Compound effects of government interventions across fields of GI.

	(12)	(13)	(14)	(15)	(16)	(17)	(18)
Variables	*TRANSPORT*	*WASTE*	*ENERGY*	*ALTER*	*DESIGN*	*AGRI*	*NUCLEAR*
*ER*	−1.971	3.515 *	1.869	1.905	0.214	2.010	0.0391
	(1.480)	(2.110)	(1.811)	(1.810)	(1.131)	(1.691)	(0.608)
*ER* × *SUB*	0.00849	0.300	−0.0264	0.455 **	−0.0231	0.168	0.0543
	(0.154)	(0.215)	(0.177)	(0.181)	(0.113)	(0.173)	(0.0689)
*ER* × *TAX*	−1.141 ***	−0.693	−0.227	−1.468 ***	−0.245	−0.425	−0.138
	(0.348)	(0.526)	(0.420)	(0.445)	(0.295)	(0.405)	(0.168)
*ER* × *UEI*	0.309 ***	0.565 ***	0.568 ***	0.478 ***	0.228 ***	0.428 ***	0.0171
	(0.0983)	(0.146)	(0.121)	(0.133)	(0.0826)	(0.104)	(0.0365)
*SUB*	−0.0895	−0.407 ***	−0.242 **	−0.534 ***	−0.0234	−0.224 **	−0.0579
	(0.0882)	(0.135)	(0.107)	(0.116)	(0.0650)	(0.110)	(0.0406)
*TAX*	0.689 ***	0.397	0.172	0.804 ***	0.252	0.321	0.173
	(0.205)	(0.329)	(0.255)	(0.273)	(0.177)	(0.260)	(0.118)
*UEI*	−0.144 ***	−0.201 ***	−0.262 ***	−0.184 ***	−0.117 ***	−0.189 ***	−0.0130
	(0.0468)	(0.0749)	(0.0548)	(0.0676)	(0.0379)	(0.0516)	(0.0179)
*FDIPC*	0.285 ***	0.289 ***	0.297 ***	0.277 ***	0.257 ***	0.218 ***	0.122 ***
	(0.0666)	(0.0835)	(0.0921)	(0.0758)	(0.0576)	(0.0689)	(0.0310)
*POP_DEN*	1.643 ***	1.716 **	2.079 **	1.522 **	1.705 **	1.999 **	1.278 *
	(0.609)	(0.807)	(0.866)	(0.772)	(0.659)	(0.999)	(0.752)
*FIRM*	−0.0471	0.265 *	0.130	0.214	−0.0889	0.0408	−0.0371
	(0.147)	(0.151)	(0.138)	(0.142)	(0.133)	(0.131)	(0.0856)
*EMPLOY*	0.0124 *	0.0315 ***	0.0218 ***	0.0283 ***	0.00791 *	0.00976	−0.000301
	(0.00632)	(0.00731)	(0.00727)	(0.00679)	(0.00413)	(0.00613)	(0.00241)
*Constant*	−0.256	−4.388 ***	−3.823 ***	−3.725 ***	−0.971	−2.694 **	−0.434
	(0.853)	(1.352)	(1.153)	(1.165)	(0.733)	(1.146)	(0.448)
City Fixed Effects	YES	YES	YES	YES	YES	YES	YES
Time Fixed Effects	YES	YES	YES	YES	YES	YES	YES
Observations	1429	1429	1429	1429	1429	1429	1429
R^2^	0.243	0.455	0.387	0.454	0.240	0.277	0.123
Adjusted R^2^	0.238	0.451	0.383	0.450	0.235	0.272	0.117

Robust standard error in parentheses; *** *p* < 0.01, ** *p* < 0.05, * *p* < 0.1

**Table 5:** Robustness checks by re-estimating the replaced dependent variable.

	(19)	(20)	(21)	(22)
Variables	NGI	OI	NGI	OI
*ER*	3.877 ***	4.096 ***	3.237	3.658 *
	(0.333)	(0.340)	(2.055)	(2.182)
*ER* × *SUB*			0.361 *	0.382 *
			(0.197)	(0.214)
*ER* × *TAX*			−1.200 **	−1.178 **
			(0.574)	(0.567)
*ER* × *UEI*			0.621 ***	0.630 ***
			(0.163)	(0.162)
*SUB*	−0.357 ***	−0.381 ***	−0.569 ***	−0.606 ***
	(0.0376)	(0.0402)	(0.125)	(0.135)
*TAX*	−0.0939	−0.0807	0.676 *	0.676 *
	(0.110)	(0.115)	(0.355)	(0.355)
*UEI*	0.0947 ***	0.121 ***	−0.206 ***	−0.183 **
	(0.0219)	(0.0228)	(0.0792)	(0.0808)
*FDIPC*	0.284 ***	0.275 ***	0.262 ***	0.254 ***
	(0.0601)	(0.0691)	(0.0573)	(0.0668)
*POP_DEN*	1.829 **	1.848 **	1.768 **	1.785 **
	(0.761)	(0.810)	(0.731)	(0.779)
*FIRM*	0.238 *	0.264 *	0.227 *	0.253 *
	(0.137)	(0.142)	(0.134)	(0.139)
*EMPLOY*	0.0317 ***	0.0363 ***	0.0303 ***	0.0350 ***
	(0.00640)	(0.00667)	(0.00618)	(0.00646)
*Constant*	−4.110 ***	−4.308 ***	−3.438 ***	−3.762 ***
	(0.469)	(0.498)	(1.292)	(1.390)
City Fixed Effects	YES	YES	YES	YES
Time Fixed Effects	YES	YES	YES	YES
Observations	1429	1429	1429	1429
R^2^	0.525	0.535	0.538	0.546
Adjusted R^2^	0.523	0.532	0.535	0.542

Robust standard error in parentheses; *** *p* < 0.01, ** *p* < 0.05, * *p* < 0.1.

## Data Availability

All data can be obtained by email from the corresponding author.
